# An Exploration of Hiking Risk Perception: Dimensions and Antecedent Factors

**DOI:** 10.3390/ijerph16111986

**Published:** 2019-06-04

**Authors:** Shengxiang She, Yunzhang Tian, Lin Lu, Iveta Eimontaite, Ting Xie, Yan Sun

**Affiliations:** 1 School of Business, Guizhou University of Finance and Economics, Guiyang 550025, China; shengxiangs@mail.gufe.edu.cn (S.S.); lulin355@163.com (L.L.); 2 School of Business, Guilin Univesity of Technology, Guilin 541004, China; 3 Center for Behavior Decision, Shannxi University of Technology, Hanzhong 723000, China; yztian@snut.edu.cn; 4 Sheffield Robotics, The University of Sheffield, Sheffield S1 3JD, UK; i.eimontaite@sheffield.ac.uk; 5 Department of Tourism Management, Beijing Institute of Petro-chemical Technology, Beijing 102617, China; xieting@bipt.edu.cn; 6 Key Laboratory of Behavioral Science, Institute of Psychology, Chinese Academy of Sciences, Beijing 100101, China

**Keywords:** hiking, risk perception, sensation seeking, China

## Abstract

Hiking is a form of green tourism which deserves promotion and popularization, especially in present day China. However, the risks inherent in hiking could have a negative impact on the development of hiking tourism. It is important to better understand how people perceive the risks of hiking and what type of experience attributes they prefer. However, no studies have investigated the nature of risk perception from the perspective of hikers. This study explores the dimensions of the perceived risk of hiking and investigates the associated factors of hiking risk perception as well as hiking preference. A questionnaire with 18 items was used to capture people’s perception of hiking risks, and two groups of samples were surveyed. Generally, this study identified two dimensions of perceived risk towards hiking based on a sample of hikers, i.e., physical risk and psychological risk. Demographic variables such as gender, upbringing background, and hiking frequency were shown to predict hiking risk perception while gender and hiking frequency predicted route preference. The personality trait of sensation seeking appeared to be a significant predictor of hiking preference. These findings lend themselves to market segmentation and marketing strategies on hiking tourism.

## 1. Introduction

About one decade ago, hardly anyone imagined the tremendous speed at which tourism would expand and grow in China. Today it has become a global phenomenon and is one of China’s leading industries. Within the tourism industry, the fastest growing sector is that of green tourism, or ecotourism, which has received a great amount of attention in recent years [1]. In 1983, the Mexican landscape architect and environmentalist Héctor Ceballos-Lascurain defined eco-tourism as environmentally responsible travel and visitation to relatively undisturbed natural areas, to enjoy and appreciate nature (and any accompanying cultural features—both past and present) that promotes conservation, has low visitor impact, and provides for beneficially active socioeconomic involvement of local populations [2]. Green tourism differs from mass tourism by having a lower impact on the environment and not requiring overly developed infrastructure. Ecotourism stresses the need to conserve the natural environment, thus offering an opportunity to capitalize on underdeveloped natural attractions without incurring the adverse effects of conventional mass tourism. Among all green tourism activities, hiking was remarkably welcomed by city dwellers in China and has become a popular way of entertainment. In 2016, there were about 18 million hikers and about 1000 domestic outdoor clubs in China [3]. 

While seeking to reduce and control risks in the workplace, modern people are, on the other hand, keen on adventure in their leisure life. By participating in adventure, and exciting, challenging outdoor hiking activities, humans exhausted by industrial civilization can release their emotions, and recover the peacefulness of body and mind [4]. However, the risk is a double-edged sword for hikers as we can see outdoor accidents occur from time to time which overshadow the benefits of the hiking tourism industry [5]. To sustain or enhance this trend of hiking tourism, it is important to understand the perceived risk driving people to choose to take up hiking. From a marketing perspective, it is important to better understand how consumers perceive the risks of hiking and what type of experience attributes they prefer. Improved knowledge of preferred experience attributes is an important means of helping tourism businesses to develop green tourism activity products that better fit the needs of the market, and to develop more efficient and integrated marketing communication strategies.

This study was expected to contribute to two aspects of investigating risk perception of hiking by focusing on Chinese tourists going hiking. First, we explore the dimensions of the perceived risk of hiking. Second, we investigate the antecedent factors of hiking risk perception as well as hiking preference. Especially, we investigate the relationship between sensation seeking and preference of hiking route types. The main object of this study is hikers, however, in order to enrich this research, we also included a college student group who had little or no experience of hiking to explore whether there are some similarities and differences between college students and social groups.

### 1.1. Perceived Risks in Tourism

Travel risk is defined as the possibility of suffering from all kinds of misfortunes while the tourist is on their trip or in tourist destinations [6]. Compared to physical goods, travel services have higher risk; therefore, risk perception is a key factor that can help to explain tourism consumer behavior and affect tourism decision making [7]. Moreover, risk associated with travel destinations is multidimensional in nature [8]. Tourist perception of multiple risk dimensions mainly refers to negative consequences that may occur during travel [9]. Therefore, researchers have paid considerable attention to find out, assess and evaluate the risk dimension associated with the tourism destinations [10,11,12,13]. 

Reisinger and Mavondo [11] presented 13 perceived risk of tourism and found the country’s culture, traveler’s personality, and motivation to have a significant relationship with risk perception (Table 1). Fuchs and Reichel [14] compared the perceived risks of different destination between travelers from different countries and then concluded that perceived risk of a destination is a multidimensional concept that varies according to the tourist’s nationality and culture. Based on a post-disaster case in Japan, Chew and Jahari [15] proposed three factors: personal risk, financial risk, and performance risk. Taking international travelers as the research object, Rita, Carmelo, and María [12] proposed health risk, risk of suffering from crime and delinquency, accident risk, environmental risk and risk from disasters. In Lu’s [13] study of the impacts of perceived risk in senior travel, there are three factors: expectation risk, socio-political risk, and physical harm risk. Among related research in a Chinese context, Xu, Xu, and Wang [16] studied the tourism perceived risk dimensions on Chinese people and identified nine dimensions. It is noteworthy that most of the existing research background is on international tourism, and the objects of study are traveling in general. Therefore, although their research conclusions have universal significance, considering the diversity and difference of various tourism scenarios, research for a specific type of tourism is still needed. For example, Zhang [17] investigated six perceived risk factors specific to sojourner traveling (Table 1) on overseas students. Among these investigated risks, sojourners were mostly concerned about personal safety risk, while they were concerned less about the social-psychological risk. Feng and Yan [4] surveyed tourists in the ancient city Pingyao on their risk perception, and the results of this study revealed three main dimensions of risks; personal risk, financial risk, and performance risk (Table 1). 

Previous studies on the classification of perceived traveling risk are helpful to understand the behavior of tourists. However, these studies lack more detailed investigation on the subjective side of risk perception. Existing tourism studies tend to disconnect rather than incorporate different cognitive and affective concepts related to risk perception, resulting in many inconsistent definitions of risk perception in tourism [20,21]. Risk perception is socially constructed and mutually shaped by the tourist and others, in that the tourists will still perceive the risks even if the real threat is eliminated. Peters, Slovic, Hibbard, and Tusler [22] proposed that risk is perceived and acted on in two fundamental ways: risk as a feeling and risk as an analysis. Therefore, risk perception is more than a perceived calculation of negative probabilities, but also a feeling of worry, anxiety, fear, etc. Yang and Nair [21] analyzed the relationship chains of concepts related to risk perception and stressed that concept of worry is a more suitable operational definition of risk in tourism than the concept of probability. Worry is the cognitive response to uncertainty, whereas uncertainty is related to perceived risk but with unknown probability [23]. According to Larsen, Brun, and Øgaard [24], worry is a chain of thoughts or cognitive responses which carries negative affection over the uncertain future or outcome. Considering most of the respondents are lay-people who have limited understanding on the probabilistic definitions of risk perception, it is hard to know whether the respondents are referring to risk or feeling of worry when they are taking the survey. 

Beyond the debates on the risk perception concept, there are some key differences between hiking and traditional mass tourism in risk perception. Traditional tourists are risk averse, but adventure tourists, as the name suggests, are risk seekers. For example, some research found that perceived risk is a factor for tourists to avoid a certain tourist destination [25], but Robinson [26] found that many people in hiking and outdoor activities are willing to accept a much higher risk in hiking than the risk which is acceptable to them in their work. As a sort of adventure travel, hiking has distinct characteristics compared to traditional mass tourism. Hiking is a form of green tourism with long, vigorous walking on or off trails in the countryside usually with a group, which involves less commercial activity such as accommodations, restaurants, supporting facilities, and so on. Therefore, whether the risk perceptions of hiking and mass tourism share common dimensions or not, whether the impact of perceived risks on tourist behavior is the same or the exact opposite, or both similarities and differences coexists, are all questions to explore. 

### 1.2. Sensation Seeking and Risk Perception in Tourism

On the motivation of the adventure tourists, studies suggest that adventure tourists seek feelings of fear and excitement under risk, rather than an actual risk [27]. The question is, however, why do some people seek a more intense feeling? Zuckerman carried out a series of studies on sensation seeking (SS) based on behavioral stimulation theory [28,29]. This work provides a useful theoretical and methodological tool to understand a distinctive tourist behavior from the perspective of personality traits. Zuckerman defined SS as a trait defined by the need for varied, novel, and complex sensations and experiences and the willingness to take physical and social risks for the sake of such experience. Among the insights gained is that there are several types of SS, identified by four subscales: thrill and adventure seeking (TAS), experience seeking (ES), boredom susceptibility (BS), and disinhibition (DIS), although most of the researchers use the total SS scale score. With the SS scale, several studies found that sensation seeking and risk preferences are related. For example, those who score high in the SS trait also have a higher propensity to participate in risky activities, including high-risk sports activities [26], high-risk occupations [30], and the use of illicit drugs or racing [28]. Gilchrist, Povey, and Dickinson [31] found that adventure travelers are high sensation seekers, whereas Galloway and Lopez [32] found that sensation seekers tend to choose remote national parks to pursue the opportunity of taking challenging activities, meeting interesting people and encountering dangerous wild animals. Pizam, Fleischer, Mansfeld [33] investigated a group of Israeli students and found that people with higher sensation-seeking tendencies are more likely to become independent travelers and more inclined to participate in risky activities. Pizam, Jeong, Reichel, et al., [34] also found that there was only a moderate correlation between risk taking and sensation seeking, which indicates that sensation seeking and risk taking are not the same construct although they are related. They further suggested that sensation seekers have a different perception of risk, rather than seeking risks. 

The research reported here builds upon these previous studies by investigating: (1) How people (hikers and less experienced students) perceive risk in the context of hiking tourism? (2) What are the antecedent factors of hiking risk perception and preference? (3) What are the implications of the results for hiking tourism practitioners?

## 2. Materials and Methods

The data was gathered from two questionnaire surveys in the city of Guilin. Guilin is an internationally renowned tourism resort in China, with its picturesque waters and mountains, stones and caves, it provides unique natural geographical conditions for outdoor activities and tourism industry. In addition to attracting lots of domestic and foreign tourists hiking in Guilin, many residents are also very keen on hiking. On weekends and holidays, hikers’ footprints spread all over the wild outskirts of Guilin. Some famous hiking routes also emerged in Guilin: for example, hiking the Lijiang river is one of China’s top 10 classic hiking tracks—among other popular routes in the area. There are many outdoor clubs in Guilin, and every weekend and on holidays there are various kinds of hiking activities, vast number of hikers are walking in groups in the mountains and countryside. The most typical way of hiking is led by professional guiders with return on the same day. Although there are self-organized small-scale hiking activities, this study focuses on team-based hiking activities, which has more managerial implications.

To ensure the reliability and validity of the questionnaire, the items were primarily derived from the previous literature. In tourist literature on perceived risk, several studies have developed corresponding risk perception scales on mass tourism, which provided an item pool for the current study [7,10,11,16]. The members of the research team often take part in hiking, so we first produced 23 preliminary items based on our own experience. Subsequently, we focused on informally interviewing five hikers, who evaluated the content validity, comprehensibility, and terminology accuracy of the items respectively. We adjusted and revised the questionnaire accordingly, and finalized 18 items for the scale. Finally, we conducted a preliminary survey of 12 college students and fine-tuned some of the items to make them more in line with the language style of lay people, without changing the meaning of the items. All the items are measured on a five-point Likert-type scale, ranging from 1 (strongly disagree) to 5 (strongly agree). In the questionnaire, the participants were asked to indicate how much they worry about each item if they were to go hiking. Before the formal survey, a pilot study was taken based on 15 graduates from Guilin. 

The questionnaire is divided into three parts. The first part measures sensation seeking of the hikers. To reduce the workload of the respondents, the eight-item Brief Sensation Seeking Scale (BSSS) [35] was used. When Leep and Gibson [36] investigated the influence of SS on tourism risk perception, they summed all of the items and produced a total SS scale score. Such treatment is effective but did not distinguish between the dimensions of sensation seeking. Subscales of SS are useful to give a more detailed description as well as to reveal the distinction between the two groups. For example, the results show that the hikers group showed less sensation seeking compared to the students in ES, TAS, and DIS, while showing higher BS (see Table 2). We think it is better to use subscales of SS as in the following regressions, not all dimensions of SS are related to risk perceptions. Therefore, this study probed into the four dimensions of SS and made a comparison between student sample and hiker sample. The second part includes 18 items measure on perceived risk in hiking. All the items are measured on a five-point Likert-type scale, ranging from 1 (strongly disagree) to 5 (strongly agree). The third part asks demographic characteristics of the respondents. The study was conducted in Chinese.

The first round of the survey was conducted on hikers in the Guilin area. We approached the hikers through online social networks, such as QQ group and We-chat which are popular in China. A total of 189 respondents filled in the questions, of which 15 were excluded from the analysis due to not fully completed questions leaving 174 questionnaires in the final analysis. The age distribution was mainly from 31 to 50 years old (69.5%). Out of 174 respondents, 57.5% were female, and 67.8% had hiked more than three times over the last year. Most respondents preferred the laid-back type of route (71.3%), whereas others preferred the adventurous type of route (28.7%). In Guilin’s hiking industry, the typical practice of the club is to divide the hiking route into two kinds according to the difficulty of the hiking route. ‘Laid-back’ type means that the distance of the route will not be too long (usually within 20 kilometers), there are no very steep and difficult mountain roads, the leader is familiar with the route, and the journey is relatively easy, focusing on enjoying the scenery along the way. ‘Adventurous’ type means that the route is relatively long, difficult to walk, and lacks adequate exploration of the route, so there is the possibility of getting lost, participants need a good physical preparation.

College students are a typical market segment group. They are young, passionate, and curious, but they do not have much disposable income. The second round of survey focused on college students in Guilin. A total of 475 questionnaires were collected, of which 47 invalid questionnaires were excluded, leaving 428 questionnaires in the final analysis. The mean age was 20.18 (SD = 0.55). Females made up a larger portion of the sample (55%), most of the respondents had a background of childhood living in a rural area (73.6%), and most of the respondents had went hiking at least one time (61%). Most respondents preferred the laid-back type of route (77.6%), whereas 22.4% preferred the adventurous type of route.

## 3. Results

### 3.1. Data Description

#### 3.1.1. Sensation Seeking

Each dimension of the SS scale contains two items for which the sum of scores is calculated respectively (Table 2). As a result, the range of SS is from 0 to 10. The internal consistency of the BSS was tested using Cronbach’s alpha (*α* = 0.862) and the reliability coefficient of each subscale is higher than the 0.70. The KMO (Kaiser-Meyer-Olkin) values were 0.935 suggesting a good validity of data.

Statistically, the two groups are different in four dimensions of sensation seeking. The students group appeared to be highly sensation seeking, as their reported means are higher than the neutral value (i.e., 6, corresponding to moderate) in three dimensions, i.e., experience seeking (ES), disinhibition (DIS), and boredom susceptibility (BS). In contrast, the hikers showed less of sensation seeking compared to the students in ES, TAS, and DIS, while showing marginally higher BS (Table 2). Besides, BS is the strongest personality trait for both student group and hiker group, which is a trait to feel extremely tired of any repetition of experience, fixed environment, tedious work, or boring people. There was no significant difference between gender, except that men had a higher DIS than women (*M* = 6.5 > 5.51, *p* ≤ 0.001).

#### 3.1.2. Perceived Risk of Hiking

Overall, students and hikers showed some concerns about the risk of hiking, and most means of the perceived risks are higher than the neutral value (i.e., three corresponding to ‘moderate’) (Table 3). Besides, they were consistent with perceptions of what is most worrying and what is least worrying. For example, the most prominent perceived risks were coincided to be “public security problems”, “traffic accident”, “outdoor medical assistance”, “poor communication”, and “sudden bad weather”. The least prominent perceived risks are “feel uncomfortable under unfamiliar surroundings”, “get lost during hiking”, “terrible team leader organization and management”, “physical exhaustion”, and “fever, acute disease”.

### 3.2. Reliability and Exploratory Factor Analysis

We used IMB SPSS Statistics 23 software to analyze the reliability of our data. The Cronbach’s α was 0.94 for the student’ sample and 0.92 for the hiker sample in perceived hiking risks, which gave an excellent data reliability. Principal components analysis was applied to the exploratory factor analysis. The KMO values of our sample data were 0.946 and 0.950 respectively (Table 4), which indicates the suitability of our data for a principal components’ analysis. 

The use of rotation factor analysis based on principal component and maximum variance extracted three common factors for students’ sample, and two factors for hikers with eigenvalue greater than 1 (Kaiser criterion) (Table 4). These factors explained 63.25% and 66.30% of the total variance respectively, implying a good explanatory power. Table 4 shows the reliability of the test results. The comprehensive reliability of each potential variable is above 0.70. The factor structure is clear, the load factor on the value is greater than 0.60 for most of the items, and the cross-measure factor is below 0.50 for most of the items.

From the literal meaning of words, the perceived risk items in the first factor of the hiker group reflect concerns about safety, so we named it ‘physical risk’. The second factor primarily reflects concerns about the negative psychological experience, so we named it ‘psychological risk’. Physical risk is the possibility of physical danger, injury, or sickness related to the safety during hiking, whereas the psychological risk is the possibility of disappointment, discontent, or other negative feelings related to psychological loss during the hike. The students and hikers are consistent regarding the psychological risk factor, but for students, the physical risk perception is broken down into two factors. The first factor primarily reflects concerns about personal safety, therefore we named it ‘personal safety risk’. The second factor reflects concerns about the external environmental incidents which will have an indirect impact on safety, so named it ‘environmental safety risk’. It must be mentioned that although the item ‘insufficient and inadequate peers would affect the team’ loaded higher on the environmental risk factor (.5), by meaning it belongs to psychological risk. Considering the factor loading 0.5 and 0.496 are not much different, so we placed this item on the psychological factor. 

### 3.3. Antecedent Factors of Perceived Risk of Hiking

To determine the antecedent factors on hiking risk perception, multiple regression models based on two samples were estimated. In the student sample, we included gender, hiking experience (operationalized as has participated or has not participated in hiking) and upbringing background as predicting variables. In the hiker sample, we included gender and hiking frequency as predicting variables. Gender is widely used as an important demographic variable in risk perception research [36,37]. Hiking experience is related to familiarity and expertise as two dimensions of prior knowledge which has long been recognized as an important factor in decision-making processes [38]. As to the upbringing background, it is logical to regard people who grew up in the countryside to have a different risk perception towards hiking. For the same reason, these predictors were included when we investigated hiking preference.

Gender has a significant association with the perceived personal safety risk (*B* = −0.456, *SE* = 0.177, *p* < 0.001) and perceived environmental risk (*B* = −0.221, *SE* = 0.127, *p* < 0.05) for the student sample. Gender also has a significant association with the perceived physical risk (*B* = −0.319, *SE* = 0.161, *p* = 0.05) for the hiker sample. It means that women perceived higher physical risk than males did. It is noteworthy that there is no gender difference on perceived psychological risk for both groups (Students: *B* = −0.064, *SE* = 0.127, *p* = 0.476; Hikers: *B* = −0.158, *SE* = 0.133, *p* = 0.30).

For students, their ‘upbringing background’ only had a significant association with the perception of personal safety risk (*B* = 0.335, *SE* = 0.138, *p* = 0.001) and hiking experiences only had a negative association with the perception of psychological risk (*B* = −0.364, *SE* = 0.146, *p* < 0.001), that is, the students who went hiking became less worried about a negative psychological experience compared to the students who never go hiking. For hikers, ‘hiking frequency’ only predicts psychological risk perception (*B* = −0.272, *SE* = 0.142, *p* < 0.05), that is, the more times they went hiking, the lower scores they had on negative psychological experience.

### 3.4. Antecedent Factor of Hiking Preference

To investigate how various factors predict an individual’s preference of hiking routes, i.e., preferring a laid-back type route or adventurous type route, we ran logistic regressions on the route type preferences (laid-back type = 1 vs. adventurous type = 0) based on two samples respectively. In the student sample, we included gender, hiking experience, SS, perceived risks, and upbringing background as predicting variables. In the hiker sample, we included gender, hiking frequency, SS, perceived risks as predicting variables. The correlations of SS with perceived hiking risks were between 0.03–0.26. The results of this logistic regression are shown in Table 5.

Firstly, gender predicts the route type preference of hiking. For both groups, males are more inclined to the adventurous type of route, whereas women tend to prefer the relaxing, laid-back type of route. For hikers, the more often they are going on a hike, the more likely they are inclined to choose adventurous routes. However, the upbringing background and hiking experiences had no association with student’s route preference.

Secondly, SS is related to the preference for hiking routes. For students, the TAS and DIS had a significant association with the hiking preference, i.e., the ones who scored higher in TAS and DIS were more likely to take an adventurous type of route in hiking. However, these findings did not apply to hikers, for whom only the ES had a considerable association with the route type preference.

Thirdly, perceived risks influence the preference of hiking routes. For the hiker group, the perceived psychological risk had a significant negative association with the route type preference, that is, the higher the perceived psychological risk, the more inclined the hiker was to an adventurous route. Also, the perceived physical risk made the respondents inclined to a laid-back type of route, but the relationship is not significant. For students, only the physical safety risk had a marginal association with hiking routes preference, i.e., the higher the personal safety risk perception, the more inclined to the laid-back type of routes.

## 4. Discussion

The purpose of this study was to develop a better understanding of the risk perception associated with hiking tourism in China. We used a questionnaire of hiking risk perception which was comprised of 18 items and we surveyed two participant groups: experienced hikers and less experienced students. This study identified two dimensions of perceived risk towards hiking based on the sample of hikers, i.e., physical risk and psychological risk. Differing from other relevant research on tourism risk perception which identified many risk factors [7,10,11,16], this study only identified two or three dimensions of perceived hiking risk. This distinction may be ascribed to the characteristics of hiking activities. Compared to traditional tourism, hiking is simple and easy, and a hike can usually be completed in a single day. Consequently, perceived hiking risks are more concentrated on fewer factors.

Contrary to our expectations, the hiker and student groups had an inconsistent structure of the perceived risk of hiking. For the student group, the physical risk is further split into two factors, i.e., the personal safety risk and the environmental safety risk. This can be explained by the socio-demographic structure of our participant groups. The main hikers in Guilin are office workers, housewives, and retirees. Normally, hikers have to pay a certain fee to the club to cover car rental, guides salaries, and insurance. In our sample, the hikers had been deeply involved in hiking activity, while most students did not participate in hiking or had a limited experience of it. Probably, different experiences shaped the distinct structures towards the risk perception of hiking. In fact, many students never went hiking, and their perceived risk of hiking mainly came from the media and imagination. In recent years, with the increasing popularity of hiking in China, an increased number of media reports about hiking activities and accidents are reported and spread widely due to the mobile internet, making this information easily accessible to college student population. Informal interviews with some students from the sample confirmed this supposition. On the contrary, the hikers group had firsthand experience of the risks involved in hiking. For them, hiking risk is either reflected in a tangible loss or in an intangible psychological loss. Nevertheless, the two groups were consistent in their perception of specific risks. Both participant groups considered ‘public security problems’, ‘traffic accident’, ‘outdoor medical assistance’, ‘poor communication’ and the ‘sudden bad weather’ as top risks. Furthermore, both groups also considered ‘feel uncomfortable under unfamiliar surroundings’, ‘get lost during hiking’, ‘terrible team leader’ organization and management’, ‘physical exhaustion’, and ‘fever, acute disease’ as least risky.

Female respondents had a higher perceived risk on the physical aspect and were more inclined to take laid-back routes than male respondents for both groups, which suggest that women are more worried about safety during hiking. For hikers, the more times they participated in hiking, the less worried they were about poor psychological experiences and they were more inclined to take an adventurous route. This finding is consistent with the intuition that people are always seeking novel experiences in tourism. Similarly, college students who went hiking worried about a negative psychological experience less compared to the counterparts without a hiking experience. Compared to the structured, well accommodated traditional tourism, hiking is particularly rich in novel, thrilling, and adventurous experiences rather than material enjoyment. Only those who have participated in it can experience it. College students who grew up in towns and cities perceived a higher risk of personal accidents than those who grew up in rural areas. The reason might be that rural people are more familiar with the wild environment than urban people.

Sensation seeking (SS) is a personality trait associated with the need for novelty and stimulation and has been linked to tourist behavior [36]. In this study, we found the student group contained more strong sensation seekers, which was reflected in three dimensions of SS. Surprisingly, the hiker group appeared to have higher scores only in one dimension of SS, i.e., boredom susceptibility. For the other three dimensions, the average scores reported were below the neutral value. The SS trait predicted hiking route preference for both hikers and students, yet slightly differently across groups. Specifically, hikers with higher scores on ES were more inclined to take an adventurous route, whereas the students scoring higher on TAS or DIS were more inclined to take an adventurous route. The results of this study confirm those of Eachus [39], and Lepp and Gibson [36] in that tourist preferences appear to be related to SS with sensation seekers favoring holidays rich in stimulation and places perceived as risky more than those lower in the SS trait. However, our study not only confirmed the relationship of SS with tourist preferences in a Chinese context but also indicated significant group differences when exploring the sub-traits of SS.

For both groups, there is a non-significant (but marginal) positive relationship between perceived physical risk (or personal safety risk) and route preferences. We suggest that the higher the perceived physical risk or personal safety risk is, the more the individual is inclined to a laid-back type of hiking route. Surprisingly, there was a significant negative relationship between the psychological risk perception and route preference for the hiker group: the ones who perceived a higher psychological risk were more inclined to take an adventurous route. This finding is contrary to the traditional understanding of tourist behavior, such as perceived risk impeded tourists [7], and the higher the level of perceived risk of tourists meaning a greater likelihood they would feel the insecurity of the environment and then withdraw [25]. It is conceivable that a hiker may worry about feeling negatively during the hike, but still prefer to pursue an adventurous route. The higher the expectations of hikers are, the more worried they will be about the experiences not living up to expectations, therefore the perceived psychological risk increases. While with higher expectations, the hikers will pursue more adventurous routes, as hikers expect novel, thrilling, and stimulating experiences through taking an adventurous route. They may rationalize the risk of hiking as it can provide the additional rewards of social acceptance and prestige [40]. Rationalizing risk is consistent with what Robinson [26] suggested in his model of enduring risk recreation involvement that perceived risk, competence in the activity, and anticipated outcomes would be part of the decision-making associated with participation in a particular experience. Similarly, adventurous routes are more challenging, and the hikers are more unfamiliar with the surroundings and have higher requirements for the ability and quality of guides and peers (i.e., items that constitute psychological risk factors). Therefore, if hikers are more worried about these aspects, which indicated a higher perceived psychological risk, it shows that they prefer more adventurous routes.

## 5. Conclusions

This study explored hiking risk perception and identified two dimensions of perceived risk towards hiking based on the sample of hikers—i.e., physical risk and psychological risk—while also identifying three dimensions of perceived risk towards hiking based on the sample of students—i.e., physical risk, environmental safety risk, and personal safety risk. The higher the perceived physical risk is, the more the individual is inclined to a laid-back type of hiking route. However, there was a significant negative relationship between the psychological risk perception and route preferences for the hiker group, which means the ones who perceived a higher psychological risk were more inclined to take an adventurous route.

### 5.1. Contributions

To the best of our knowledge, our research is the first to explore the dimensions of perceived risk towards hiking. Consumers’ perceived risks are the key factors that influence consumer travel decision [16]. It is especially important to identify dimensions of the perceived risk and to study the driving force. Although several researchers investigate the structures of perceived travelling risk in general, our research found that the perceived risks of hiking can be reduced into two or three dimensions, i.e., physical risk and psychological risk. We also investigated the factors’ associations with the risk perception and preference of hiking. The demographic variables such as gender, upbringing background, and hiking frequency predicted hiking risk perception while gender and hiking frequency predicted route preference. Besides, we investigated the personality trait of sensation seeking which was a significant predictor of hiking preference. One of the strengths of this study compared to other studies that have addressed tourism, risk, and SS is that in addition to college student sample, we have included experienced hikers.

### 5.2. Management Implications

In terms of practice, these findings lend themselves to market segmentation, marketing strategies, and a better understanding of who is likely to go hiking instead of packaged tourism. With increased income and hedonistic pursuit, more and more Chinese people are keen on traveling. However, the vigorous development of the tourism industry in China brings heavy environmental problems; there is an urgent need to transform traditional tourism into green tourism. Although hiking is getting popular among city dwellers as a form of green tourism, the risks inherent in hiking emerge at the same time. The findings of this study suggest that the perceived risk of hiking is a multi-dimension concept which requires marketers to understand better that risk is a double-edged sword for attracting hikers. Multiple types of hiking routes should be designed to meet the needs of diverse groups of people, and the risk level of the routes should be quantified to enable informed decision-making. In the minds of many people, hiking is a very tiring and risky activity, whereas in other people’s eyes that is the temptation. However, what hiking-lovers seek is the unique feeling of stimulation, excitement, even fear, but not the unnecessary risk [36]. After all, sensation seeking does not equate with a “death wish” [29]. The marketers must consider both the reality and the art of hiking tourism and find a balance between a stimulating activity and risk management so that hikers can get the best experience under the premise of ensuring safety.

This research suggests gender, hiking frequency, as well as SS would be a useful segmentation tool. For example, marketers should highlight the risk control measures to reduce the perceived risk for women whereas stress the novelty and route challenges to attract the regular hikers. It might be best to design separate promotional messages for high sensation seekers and low sensation seekers. For example, the primitiveness and adventure often marketed as part of an ecotourism package may attract sensation seekers while discouraging those low in SS. Lepp and Gibson [36] suggested that the same package could be presented as an opportunity to participate in the conservation of an ecologically valuable area to appeal to some lower in SS.

### 5.3. Limitations and Future Directions

Like any research, the current study suffers from some limitations, which should be kept in mind when making conclusions on our findings. First, although the results reported in this study indicate that a relationship between risk perception and hiking preference exists, the strength of this relationship needs further investigation. Second, this study concentrated on the factor structure of perceived risk. Future research needs to address the external validity of the questionnaire. Third, our samples were restricted to hikers and college students in Guilin, China. Hikers may view risk issues differently due to the differences of geography and culture. Future research needs to evaluate the generalizability of the study result to broader areas. Going forward, the structure and content of perceived hiking risk provide a basis for exploring its consequence variables, such as the effects of hiking risk perceptions on revisit intentions as well as risk reduction strategy. Future studies will focus on hiking behavior from a risk perspective.

## Figures and Tables

**Table 1:** Dimensions of travel risk.

Studies	Dimensions
Moutinho [10]	Economic risk, physical risk, the risk of natural disasters, health risks, psychological risks, terrorism risks, social risks, and criminal risk
Roehl and Fesenmaier [7]	Transportation, law, order, health, accommodation, weather, tourist attractions, and medical assistance
Reisinger and Mavondo [11]	Culture, equipment, finances, health, physical, political, psychological, satisfaction, social, hijacking, explosion, biological attack, and time.
Xu, Xu, and Wang [16]	Physical risk, functional risk, financial risk, communication risk, psychological risk, social risk, service risk, facility risk, and communication risks
Zhang [17]	Personal safety risk, traffic risk, financial risk, functional risk, cultural risk, social-psychological risk
Feng and Yan [4]	Personal risk, financial risk, and performance risk
Chew and Jahari [15]	Personal risk, financial risk, and performance risk
Casidy and Wymer [18]	Financial risk, social risk, performance risk, and psychological risk
Cui et al. [9]	Human risk, psychosocial risk, food safety, weather risk, finances, quality of service, natural disasters, and accidents
Rita, Carmelo, María [12]	Health risk, risk of suffering from crime and delinquency, accident risk, environmental risk, and risk from disasters
Susanto, Susatyo, Rizkiyah et al. [19]	Perception of serious accident, perception of the probability of accident event mountaineering activity
Lu [13]	Expectation risk, socio-political risk, physical harm risk

**Table 2:** Description and mean comparison of SS (sensation seeking).

SS Dimension	Students		Hikers		Mean Comparison	
	M	SD	M	SD	*t*	*p*
ES	7.01	2.05	5.69	2.16	5.50	<0.001
BS	7.44	1.86	7.71	1.65	−2.39	0.072
TAS	5.90	2.00	4.88	1.87	5.72	<0.001
DIS	6.74	2.09	5.93	1.79	3.95	<0.001

Note: M = mean; SD = standard deviation; ES = experience seeking; BS = boredom susceptibility; TAS = thrill and adventure seeking; DIS = disinhibition.

**Table 3:** Description of hiking risk perception.

Items of Perceived Risks (PR) I Worry about:	Hikers		Students	
	M	SD	M	SD
PR1: sudden bad weather (rain, lightning, snow, fog, heat, etc.)	3.43	1.01	3.42	1.13
PR2: encountering public security problems on the way	3.36	1.01	3.46	1.06
PR3: traffic accidents	3.34	1.08	3.38	1.13
PR4: outdoor medical assistance is not helpful when in need	3.33	1.00	3.36	1.07
PR5: poor communication with the outside world	3.33	1.00	3.41	1.13
PR6: wildlife invasion, insect bites	3.29	1.01	3.17	1.20
PR: occasional geological disasters such as earthquakes, mudslides, landslides etc.	3.28	1.16	3.15	1.28
PR8: faults in outdoor equipment and facility	3.24	1.03	3.37	1.02
PR9: accidents such as falling, slips, injuries, etc.	3.19	1.06	3.03	1.21
PR10: insufficient and inadequate peers would affect the team	3.14	0.99	3.05	1.11
PR11: loss of property during hiking	3.11	1.09	3.27	1.07
PR12: contaminated food, infectious disease	3.10	1.07	3.21	1.21
PR13: hiking experience falling short of expectations	3.09	0.92	2.97	1.01
PR14: getting fever or acute disease during hiking	3.03	1.10	2.96	1.18
PR15: physical exhaustion	3.03	0.99	2.98	1.25
PR16: inadequate the team leader’ organization and management	3.01	1.07	3.14	1.01
PR17: getting lost during the hike	3.00	1.05	3.13	1.21
PR18: feeling uncomfortable in unfamiliar surroundings	2.49	0.97	2.89	1.16

Note: M = mean; SD = standard deviation.

**Table 4:** Factor structures of perceived risks of hiking.

Item	Hikers		Students		
	Physical Risk Cronbach’s α = 0.959	Psychological Risk Cronbach’s α = 0.822	Personal Safety Risk Cronbach’s α = 0.903	Environmental Safety Risk Cronbach’s α = 0.908	Psychological Risk Cronbach’s α = 0.742
PR13	0.12	**0.672**	0.143	0.176	**0.766**
PR18	0.356	**0.629**	0.359	0.063	**0.742**
PR16	0.161	**0.783**	0.073	0.536	**0.585**
PR10	0.189	**0.806**	0.138	0.500	**0.496**
PR2	0.519	**0.624**	0.272	**0.726**	0.196
PR3	**0.656**	0.543	0.341	**0.792**	0.115
PR8	**0.684**	0.417	0.401	**0.743**	0.204
PR4	**0.703**	0.5	0.379	**0.745**	0.216
PR5	**0.719**	0.431	0.445	**0.609**	0.237
PR7	**0.841**	0.19	0.450	**0.539**	0.164
PR11	**0.822**	0.283	**0.608**	0.496	0.078
PR17	**0.776**	0.305	**0.686**	0.157	0.202
PR14	**0.818**	0.232	**0.649**	0.446	0.083
PR9	**0.841**	0.143	**0.765**	0.313	0.122
PR15	**0.7**	0.214	**0.731**	0.193	0.187
PR6	**0.787**	0.137	**0.721**	0.256	0.206
PR12	**0.742**	0.37	**0.592**	0.441	0.213
PR1	**0.762**	0.243	**0.649**	0.340	0.167
KMO		0.946	0.950		
Butler spherical test		<0.001	<0.001		
Total variance explained		63.25%	66.30%		

Note: The corresponding factor loadings are in bold in the table.

**Table 5:** Logistic regression on hiking preference.

Students			Hikers		
	B	*p*		B	*p*
Gender	−0.52 **	0.032	Gender	−1.26 **	0.002
Hiking experience	−0.10	0.426	Hiking frequency	−0.78 **	0.026
ES	−0.02	0.74	ES	−0.33 **	0.005
BS	0.03	0.662	BS	0.04	0.777
TAS	−0.27 ***	0.001	TAS	−0.14	0.306
DIS	−1.20 **	0.012	DIS	0.10	0.481
Psychological risk	0.03	0.816	Psychological risk	−0.42 **	0.042
Environmental safety risk	0.07	0.422	Physical risk	0.21	0.29
Personal safety risk	0.20 *	0.091			
Upbringing background	0.17	0.437			

Note: B = coeffecient of regression; ES = experience seeking; BS = boredom susceptibility; TAS = thrill and adventure seeking; DIS = disinhibition. *** indicates the significance level of 0.01, ** indicates the significance level of 0.05, * indicates the significance level of 0.1.
